# PD38 - Clinical features and natural history of cold contact urticaria in children

**DOI:** 10.1186/2045-7022-4-S1-P38

**Published:** 2014-02-28

**Authors:** Anna Pananaki, Dimitrios Mitsias, Georgios Konstantinou, Evi Delicha, Emmanouil Manousakis, Nikolaos Douladiris, Nikolaos G Papadopoulos

**Affiliations:** 1 Unit of Allergology and Clinical Immunology, 2nd Pediatric Clinic, “P. and A. Kyriakou” Children’s Hospital, Athens, Greece; 2 Department of Allergy and Clinical Immunology, 424 General Military Training Hospital, Thessaloniki, Greece; 3 Statistical Consultant, Athens, Greece

## Introduction

Cold contact urticaria (CCU) or Acquired cold urticaria is a common subtype of physical urticaria characterized by itchy wheals and/or angioedema after cold exposure. However, extensive cold contact of large skin areas may lead to systemic reactions.The prevalence and course of CCU is not well defined.

## Objective

To present epidemiological and clinical data of children with CCU, natural progression and to examine this progression according to the cold stimulation test (CST).

## Methodology

Data were collected from 32 children with cold urticaria≤18 years old who have been evaluated at the Allergy Unit of PandA Kyriakou Children’s Hospital. Demographic data, personal history, laboratory testing, cold stimulation testing (CST), atopy assessment, medication and disease severity were recorded. Telephone interviews of patients and/or their parents, or annual consultations were performed in order to obtain follow up data.

## Results

We excluded 2 patients because secondary causes were found. The most common trigger was swimming in the sea. History for atopy was present only in 37% of patients.The mean age of onset was 9.2±3.4 years old. Roc analysis for CST with respect to the presence of prior anaphylaxia revealed that the optimal cut off for the test was the 3 minutes. This cut off, presented a sensitivity of 90% and a specificity of 82.4%. The AUC was 0.826, 95%CI:[0.636-1].The median duration of surveillance was 70,4 months. During this time interval 17% showed persistent or even worsening symptomatology, 27% reported improvement, while 30% overcame their symptoms; we had no current data for 27%. There was a trend for an association of the CST with disease outcome; however, the numbers were small for a conclusion.

## Conclusions

CCU in children is a chronic persistent disorder. The CST is a good predictor for anaphylaxis. It is currently difficult to predict when and in which patient the symptoms will resolve.

